# Personal Notice

**Published:** 1864-03

**Authors:** 


PERSONAL NOTICE.—Dr. J. Andrews' Post Office address is hereafter Jamestown, Chautauqua county, N. Y.

